# LIPOEDEMA IN AN ADOLESCENT MALE FOLLOWING VALPROATE DISCONTINUATION: A CASE REPORT

**DOI:** 10.2340/jrm.v58.46824

**Published:** 2026-10-02

**Authors:** Serkan KEMER, Aysel Özge KEMER, Elif Can ÖZDEMIR, Ayşegül YAMAN

**Affiliations:** 1 Department of Medical Pharmacology, Gülhane Faculty of Medicine, University of Health Sciences, Ankara; 2 Department of Physical Medicine and Rehabilitation, Gülhane Training and Research Hospital, Ankara; 3 Department of Physical Medicine and Rehabilitation, Ankara Gülhane Health Application and Research Center, University of Health Sciences, Ankara, Türkiye.

Lipoedema is a chronic adipose tissue disease characterized by symmetrical and painful enlargement of the extremities without foot involvement, easy bruising, and typically a negative Stemmer sign. Diagnosis is primarily based on clinical evaluation, with imaging methods used to support the diagnosis. Current guidelines emphasize careful evaluation of the patient’s phenotype, exclusion of lymphoedema and venous diseases, and the application of conservative approaches including exercise, compression, and complex decongestive therapy as first-line treatment (1). From a rehabilitation perspective, lipoedema is well suited to be addressed within the framework of the International Classification of Functioning, Disability and Health (ICF). In this framework, changes in body structure are evaluated in interaction with activity limitations (mobility, walking) and participation limitations, thus ensuring that treatment focuses not only on cosmetic results but also on functionality. Ultrasonography can support the diagnosis by showing increased subcutaneous fat tissue and heterogeneity (2). In contrast, lymphoscintigraphy is usually normal or non-specific and is mainly used to rule out concomitant lymphoedema.

Lipoedema is considered to be a condition that occurs almost entirely in women. To our knowledge, male lipoedema is rare, with only a small number of cases reported in the literature (3). One previously reported patient was a 62-year-old male who was initially mistakenly diagnosed with lymphoedema and treated, and later diagnosed with lipedema (4). No case in an adolescent or paediatric male, and no case describing a temporal relationship with antiepileptic drug discontinuation, has previously been reported.

Previously in this journal, it was reported that the combined use of patient-self-assessment and performance-based physical function measures in a cohort of lipoedema and lymphoedema patients provided a more comprehensive assessment compared with the use of either measure alone (5). We applied this approach here. We present this case and discuss a history of drug use that may be important in recognizing similar clinical presentations in the future.

## CASE REPORT

A 13-year-old male patient presented with complaints of symmetrical, non-pitting swelling in both lower extremities that had persisted for 3 years. The swelling mainly affected the thighs and calves, extending from the hip to the ankle, sparing the feet. The patient experienced a feeling of heaviness in the evening, tenderness, and ankle discoloration on exertion. The patient was diagnosed with epilepsy at age 5 and treated with 200 mg of valproic acid syrup twice daily for 5 years. Valproic acid (VPA) was discontinued 3 years before presentation after the seizures achieved permanent remission. Within a few months of discontinuing VPA, he gained approximately 30 kg in weight, and progressive swelling in the lower extremities developed. Subsequently, his body weight remained stable, but he had gained an additional 10 kg in the last 3 months. The patient was also given 120 µg of desmopressin at bedtime for 6 months due to nocturnal enuresis; no hyponatraemia or changes in symptoms were observed during this treatment. The patient also had thalassaemia trait without anaemia.

Physical examination revealed an obese adolescent male patient (height: 156 cm, 50^th^ percentile; weight: 83 kg; BMI: 34.1 kg/m², > 99^th^ percentile; Tanner stage: 3). The thighs and calves were symmetrically enlarged, tender, and soft; skin colour was normal. The feet and toes were unaffected, with a distinct borderline at the ankle and a negative Stemmer sign. These findings were consistent with early-stage (Stage 1) type III lipoedema. Other systemic examination findings were normal.

Lymphoscintigraphy showed normal and symmetrical lymphatic drainage without dermal backflow, thus ruling out lymphoedema. Venous Doppler ultrasonography and abdominal ultrasonography revealed no pathology in terms of venous insufficiency, thrombosis, or retroperitoneal obstruction. Laboratory investigations showed normal renal, hepatic, and thyroid function; only slight elevations in erythrocyte sedimentation rate (ESR), C-reactive protein (CRP), and lactate dehydrogenase (LDH) levels were detected. LH, FSH, and testosterone levels were within the range consistent with pubertal age, and HbA1c was normal. Ultrasonography revealed an increase in pretibial subcutaneous fat tissue thickness (1.7 cm; Fig. 1A), supporting the clinical diagnosis of lipoedema. Differential diagnoses considered included chronic venous insufficiency, limb enlargement due to obesity, systemic oedema, Dercum’s disease, and vascular malformation/overgrowth syndromes. Clinical findings, imaging methods, and laboratory results were used to exclude other conditions, and findings supporting the diagnosis of lipoedema were obtained (6–8).

**Fig. 1:**
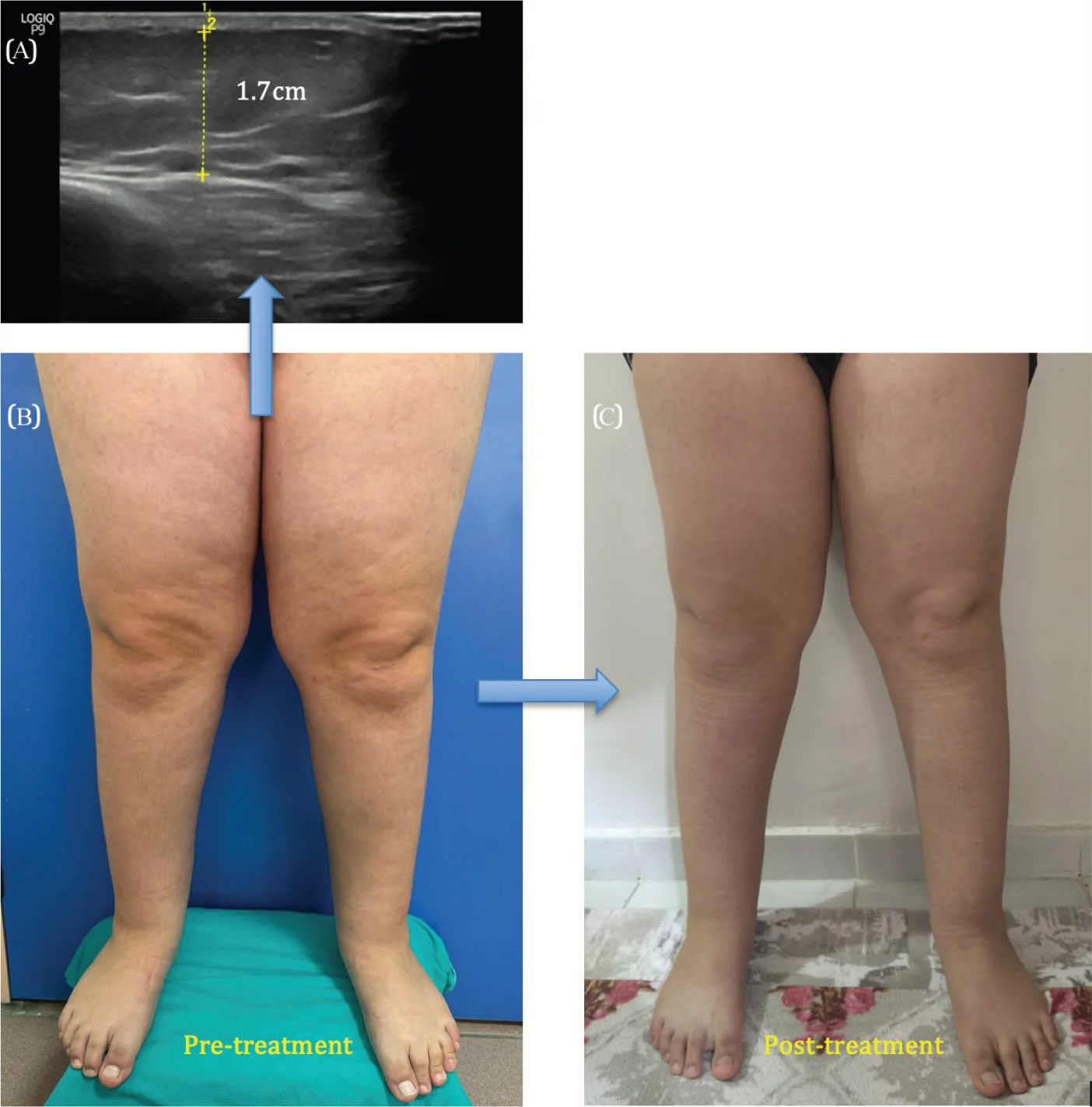
(A) Pretibial subcutaneous fat thickness measured by ultrasonography (1.7 cm). (B) Lower extremities before treatment with complex decongestive therapy (manual lymph drainage, multilayer compression bandaging, and exercise) and pneumatic compression. (C) Lower extremities after treatment, showing reduced limb circumference and volume.

In line with the ICF-based approach, outcome measures were assessed before and after treatment, encompassing body function (pain, Visual Analogue Scale [VAS], 0–10) and activity level (patient self-assessment of functionality: Lower Extremity Functional Scale [LEFS]; performance-based mobility: Timed Up and Go Test [TUG] and 30-second Sit-to-Stand test [30s-STS]). Pneumatic compression, along with complex decongestive therapy (manual lymphatic drainage, multilayer compression bandaging, and exercise), was applied 5 days a week for 3 weeks and was well tolerated. After 3 weeks, the VAS score improved from 7 to 3, the LEFS score from 51 to 72, the TUG time from 8.2 s to 5.6 s, and the 30s-STS performance from 9 repetitions to 17 repetitions. Significant improvement was also observed in walking endurance. A decrease in limb volume was detected in both lower extremities (Table I, Fig. 1B–C).

**Table I:** Clinical and limb-volume changes before and after treatment

Measure	Pre-treatment	Post-treatment (3 weeks)
VAS (0–10)	7	3
LEFS	51	72
TUG (s)	8.2	5.6
30s-STS (repetitions)	9	17
Right limb volume(cm^3^)	5,606	5,069 (↓ 9.6%)
Left limb volume (cm^3^)	5,407	5,143 (↓ 4.90%)

VAS, Visual Analog Scale; LEFS, Lower Extremity Functional Scale; TUG, Timed Up and Go; 30S-STS, 30-second sit-to-stand.

This report has been prepared in accordance with the institution’s research ethics practices. As it is a single-case retrospective report without experimental intervention, formal Institutional Review Board approval was not required. Written informed consent was obtained from the patient’s legal guardian for publication of the case.

## DISCUSSION

This case expands the known clinical spectrum of lipoedema in 2 ways: To our knowledge, it is the first case in an adolescent male and it is also the first case to describe a temporal relationship with discontinuation of an antiepileptic drug (Table II). Early, systematic differentiation from lymphoedema, enabled by careful phenotyping, lymphoscintigraphy, and venous Doppler ultrasonography, helped avoid the diagnostic delay seen in another male case reported in the literature, in which lipoedema was initially misdiagnosed and treated as lymphoedema, with the diagnosis being delayed until disease progression occurred (4).

**Table II:** Comparison of the present case with the 62-year-old male published case of lipoedema in a male patient

Feature	Bertlich et al. (4)	Present case	Comment
Age/sex	62-year-old male	13-year-old male (pubertal)	First reported adolescent case
Distribution/stage	Bilateral thigh > calf; Stage IV	Bilateral thigh and calf, hip-to-ankle (Type III); Stage 1	Present case caught at an earlier morphological stage
Prior misdiagnosis	Initially treated as lymphoedema elsewhere, with disease progression	None; lymphoedema excluded at presentation by lymphoscintigraphy	Underscores value of early lymphoscintigraphy/Doppler work-up in males
Associated factors	Type 1 diabetes, hypertension; BMI 31.4	Valproate discontinuation, desmopressin use, pubertal timing; BMI 34.1	No overlapping exposures; present case raises a novel medication hypothesis
Treatment	Three sessions of tumescent liposuction	3-week complex decongestive therapy + pneumatic compression	Reflects difference in stage; conservative care appropriate for early-stage paediatric disease
Outcome	Symptomatic improvement; 20 kg regained over 2.5 years	VAS, LEFS, TUG, 30s-STS and limb volume improved at 3 weeks	Short-term follow-up only in present case

We present the medication history as a hypothesis-generating element rather than a causal one. Valproic acid (VPA) is a histone deacetylase (HDAC) inhibitor that suppresses preadipocyte differentiation during its active period (9). We suggest that the removal of this suppressive effect may trigger a compensatory or “rebound” type of adipogenic response in susceptible tissues. VPA also disrupts mitochondrial fatty acid oxidation and triglyceride metabolism (10); it is also independently associated with weight gain through increased appetite, altered lipid utilization, decreased insulin sensitivity, and decreased adiponectin levels (11, 12). Together, these mechanisms offer a biologically consistent explanation for the observed temporal pattern.

In male patients with epilepsy, VPA can cause a decrease in FSH and testosterone levels, although its effects on LH are variable (13). As our patient is in Tanner stage 3, pubertal timing may have interfered with these hormonal changes. Furthermore, desmopressin (DDAVP), a V2 receptor agonist that increases water reabsorption in the kidneys, has also been shown to trigger endothelial von Willebrand factor release (14). While this suggests a possible microvascular contribution, we consider desmopressin to be a modulator rather than a primary cause.

The clinical picture, characterized by pain and tenderness, easy bruising, worsening of symptoms during the day, preservation of the dorsum of the feet, negative Stemmer sign, and normal lymphoscintigraphy, supported the diagnosis of type III lipoedema compared with lymphoedema or simple obesity (15). In this case, clinically significant gains were achieved in both patient self-assessment measures (LEFS) and performance-based measures (TUG, 30s-STS), supporting the use of these 2 assessment areas together in outcome measurement. This finding is also consistent with cohort results previously reported in this journal in lipoedema and lymphoedema patient groups (5).

Treatment priorities in adolescent lipoedema should focus on cardiometabolic health through activity and participation outcomes under ICF guidance, rather than solely weight loss or cosmetic appearance. In this context, within a multidisciplinary programme, supervised exercise, nutrition aimed at stabilizing bodyweight, compression therapy to the extent tolerated, and psychosocial support should be applied in collaboration with paediatricians, endocrinologists, physical medicine and rehabilitation specialists, and nutritionists (16). Given the widespread use of VPA in the treatment of paediatric and adolescent epilepsy, clinicians should be alert to unexpected changes in body composition or limb contour and should consider early rehabilitation referral when these changes are accompanied by pain or functional limitations.

Strengths include careful phenotyping, normal lymphoscintigraphy findings, and the application of a systematic differential diagnosis approach. However, the lack of longitudinal endocrinological profile monitoring, the short follow-up period (3 weeks), and the constraints imposed by drawing conclusions from a single case are significant limitations; therefore, causality could not be established. Recognizing lipoedema in adolescent males can prevent misclassification and allow for timely initiation of conservative rehabilitation treatment. Further research is needed to evaluate the potential role of desmopressin as a microvascular modulator in conjunction with the discontinuation of VPA during puberty.
